# Supplementary material for: Effects of Long-Term Supplementation of Bovine Colostrum on the Immune System in Young Female Basketball Players. Randomized Trial
Source: Nutrients. 2020 Dec 30;13(1):118. doi: 10.3390/nu13010118 (PMC7823942; doi:10.3390/nu13010118)
Supplement: Supplementary file 1 [file nutrients-13-00118-s001.pdf]

Table S1. Post-hoc test results for IL-10

Comparison	p-value
control I 3-h after - suppl I 3-h after	1.00
control I 3-h after - control II 3-h after	0.29
control I 3-h after - suppl II 3-h after	0.51
control I 3-h after - control III 3-h after	<0.001
control I 3-h after - suppl III 3-h after	<0.001
control I 3-h after - control I BEFORE	1.00
control I 3-h after - suppl I BEFORE	1.00
control I 3-h after - control II BEFORE	1.00
control I 3-h after - suppl II BEFORE	1.00
control I 3-h after - control III BEFORE	<0.001
control I 3-h after - suppl III BEFORE	<0.001
control I 3-h after - control I JUST AFTER	0.10
control I 3-h after - suppl I JUST AFTER	1.00
control I 3-h after - control II JUST AFTER	1.00
control I 3-h after - suppl II JUST AFTER	1.00
control I 3-h after - control III JUST AFTER	<0.001
control I 3-h after - suppl III JUST AFTER	<0.001
suppl I 3-h after - control II 3-h after	1.00
suppl I 3-h after - suppl II 3-h after	1.00
suppl I 3-h after - control III 3-h after	<0.001
suppl I 3-h after - suppl III 3-h after	<0.001
suppl I 3-h after - control I BEFORE	1.00
suppl I 3-h after - suppl I BEFORE	1.00
suppl I 3-h after - control II BEFORE	1.00
suppl I 3-h after - suppl II BEFORE	1.00
suppl I 3-h after - control III BEFORE	0.00
suppl I 3-h after - suppl III BEFORE	0.00
suppl I 3-h after - control I JUST AFTER	1.00
suppl I 3-h after - suppl I JUST AFTER	1.00
suppl I 3-h after - control II JUST AFTER	1.00
suppl I 3-h after - suppl II JUST AFTER	1.00
suppl I 3-h after - control III JUST AFTER	<0.001
suppl I 3-h after - suppl III JUST AFTER	<0.001
control II 3-h after - suppl II 3-h after	1.00
control II 3-h after - control III 3-h after	<0.001
control II 3-h after - suppl III 3-h after	0.00
control II 3-h after - control I BEFORE	1.00
control II 3-h after - suppl I BEFORE	1.00
control II 3-h after - control II BEFORE	1.00
control II 3-h after - suppl II BEFORE	0.51
control II 3-h after - control III BEFORE	0.02
control II 3-h after - suppl III BEFORE	0.58
control II 3-h after - control I JUST AFTER	1.00
control II 3-h after - suppl I JUST AFTER	1.00
control II 3-h after - control II JUST AFTER	1.00
control II 3-h after - suppl II JUST AFTER	1.00
control II 3-h after - control III JUST AFTER	0.00
control II 3-h after - suppl III JUST AFTER	0.02
suppl II 3-h after - control III 3-h after	<0.001
suppl II 3-h after - suppl III 3-h after	<0.001
suppl II 3-h after - control I BEFORE	1.00
suppl II 3-h after - suppl I BEFORE	0.38
suppl II 3-h after - control II BEFORE	1.00
suppl II 3-h after - suppl II BEFORE	0.09

suppl II 3-h after - control III BEFORE	0.05
suppl II 3-h after - suppl III BEFORE	0.14
suppl II 3-h after - control I JUST AFTER	1.00
suppl II 3-h after - suppl I JUST AFTER	1.00
suppl II 3-h after - control II JUST AFTER	1.00
suppl II 3-h after - suppl II JUST AFTER	1.00
suppl II 3-h after - control III JUST AFTER	0.00
suppl II 3-h after - suppl III JUST AFTER	0.00
control III 3-h after - suppl III 3-h after	0.76
control III 3-h after - control I BEFORE	<0.001
control III 3-h after - suppl I BEFORE	<0.001
control III 3-h after - control II BEFORE	<0.001
control III 3-h after - suppl II BEFORE	<0.001
control III 3-h after - control III BEFORE	<0.001
control III 3-h after - suppl III BEFORE	0.00
control III 3-h after - control I JUST AFTER	<0.001
control III 3-h after - suppl I JUST AFTER	<0.001
control III 3-h after - control II JUST AFTER	<0.001
control III 3-h after - suppl II JUST AFTER	<0.001
control III 3-h after - control III JUST AFTER	0.09
control III 3-h after - suppl III JUST AFTER	0.02
suppl III 3-h after - control I BEFORE	<0.001
suppl III 3-h after - suppl I BEFORE	<0.001
suppl III 3-h after - control II BEFORE	<0.001
suppl III 3-h after - suppl II BEFORE	<0.001
suppl III 3-h after - control III BEFORE	1.00
suppl III 3-h after - suppl III BEFORE	0.56
suppl III 3-h after - control I JUST AFTER	0.00
suppl III 3-h after - suppl I JUST AFTER	<0.001
suppl III 3-h after - control II JUST AFTER	<0.001
suppl III 3-h after - suppl II JUST AFTER	<0.001
suppl III 3-h after - control III JUST AFTER	1.00
suppl III 3-h after - suppl III JUST AFTER	1.00
control I before - suppl I BEFORE	1.00
control I before - control II BEFORE	1.00
control I before - suppl II BEFORE	1.00
control I before - control III BEFORE	<0.001
control I before - suppl III BEFORE	0.01
control I before - control I JUST AFTER	1.00
control I before - suppl I JUST AFTER	1.00
control I before - control II JUST AFTER	1.00
control I before - suppl II JUST AFTER	1.00
control I before - control III JUST AFTER	<0.001
control I before - suppl III JUST AFTER	<0.001
suppl I before - control II BEFORE	1.00
suppl I before - suppl II BEFORE	1.00
suppl I before - control III BEFORE	<0.001
suppl I before - suppl III BEFORE	<0.001
suppl I before - control I JUST AFTER	0.62
suppl I before - suppl I JUST AFTER	1.00
suppl I before - control II JUST AFTER	1.00
suppl I before - suppl II JUST AFTER	1.00
suppl I before - control III JUST AFTER	<0.001
suppl I before - suppl III JUST AFTER	<0.001
control II before - suppl II BEFORE	1.00
control II before - control III BEFORE	<0.001
control II before - suppl III BEFORE	0.17
control II before - control I JUST AFTER	1.00

control II before - suppl I JUST AFTER	1.00
control II before - control II JUST AFTER	1.00
control II before - suppl II JUST AFTER	1.00
control II before - control III JUST AFTER	<0.001
control II before - suppl III JUST AFTER	<0.001
suppl II before - control III BEFORE	<0.001
suppl II before - suppl III BEFORE	<0.001
suppl II before - control I JUST AFTER	0.21
suppl II before - suppl I JUST AFTER	1.00
suppl II before - control II JUST AFTER	1.00
suppl II before - suppl II JUST AFTER	1.00
suppl II before - control III JUST AFTER	<0.001
suppl II before - suppl III JUST AFTER	<0.001
control III before - suppl III BEFORE	1.00
control III before - control I JUST AFTER	0.05
control III before - suppl I JUST AFTER	<0.001
control III before - control II JUST AFTER	<0.001
control III before - suppl II JUST AFTER	0.00
control III before - control III JUST AFTER	1.00
control III before - suppl III JUST AFTER	1.00
suppl III before - control I JUST AFTER	1.00
suppl III before - suppl I JUST AFTER	0.00
suppl III before - control II JUST AFTER	0.00
suppl III before - suppl II JUST AFTER	0.00
suppl III before - control III JUST AFTER	1.00
suppl III before - suppl III JUST AFTER	1.00
control I just after - suppl I JUST AFTER	1.00
control I just after - control II JUST AFTER	1.00
control I just after - suppl II JUST AFTER	1.00
control I just after - control III JUST AFTER	<0.001
control I just after - suppl III JUST AFTER	0.06
suppl I just after - control II JUST AFTER	1.00
suppl I just after - suppl II JUST AFTER	1.00
suppl I just after - control III JUST AFTER	<0.001
suppl I just after - suppl III JUST AFTER	<0.001
control II just after - suppl II JUST AFTER	1.00
control II just after - control III JUST AFTER	<0.001
control II just after - suppl III JUST AFTER	<0.001
suppl II just after - control III JUST AFTER	<0.001
suppl II just after - suppl III JUST AFTER	<0.001
control III just after - suppl III JUST AFTER	1.00

Table S2. Post-hoc test results for IL-10

Comparison	p
control I 3-h after - suppl I 3-h after	1.0000
control I 3-h after - control II 3-h after	<0.001
control I 3-h after - suppl II 3-h after	<0.001
control I 3-h after - control III 3-h after	<0.001
control I 3-h after - suppl III 3-h after	<0.001
control I 3-h after - control I BEFORE	1.0000
control I 3-h after - suppl I BEFORE	1.0000
control I 3-h after - control II BEFORE	<0.001
control I 3-h after - suppl II BEFORE	1.0000
control I 3-h after - control III BEFORE	<0.001
control I 3-h after - suppl III BEFORE	<0.001
control I 3-h after - control I JUST AFTER	1.0000
control I 3-h after - suppl I JUST AFTER	1.0000
control I 3-h after - control II JUST AFTER	<0.001
control I 3-h after - suppl II JUST AFTER	<0.001
control I 3-h after - control III JUST AFTER	<0.001
control I 3-h after - suppl III JUST AFTER	<0.001
suppl I 3-h after - control II 3-h after	<0.001
suppl I 3-h after - suppl II 3-h after	<0.001
suppl I 3-h after - control III 3-h after	<0.001
suppl I 3-h after - suppl III 3-h after	<0.001
suppl I 3-h after - control I BEFORE	1.0000
suppl I 3-h after - suppl I BEFORE	1.0000
suppl I 3-h after - control II BEFORE	<0.001
suppl I 3-h after - suppl II BEFORE	1.0000
suppl I 3-h after - control III BEFORE	<0.001
suppl I 3-h after - suppl III BEFORE	<0.001
suppl I 3-h after - control I JUST AFTER	1.0000
suppl I 3-h after - suppl I JUST AFTER	1.0000
suppl I 3-h after - control II JUST AFTER	<0.001
suppl I 3-h after - suppl II JUST AFTER	<0.001
suppl I 3-h after - control III JUST AFTER	<0.001
suppl I 3-h after - suppl III JUST AFTER	<0.001
control II 3-h after - suppl II 3-h after	1.0000
control II 3-h after - control III 3-h after	0.2560
control II 3-h after - suppl III 3-h after	1.0000
control II 3-h after - control I BEFORE	<0.001
control II 3-h after - suppl I BEFORE	<0.001
control II 3-h after - control II BEFORE	1.0000
control II 3-h after - suppl II BEFORE	<0.001
control II 3-h after - control III BEFORE	1.0000
control II 3-h after - suppl III BEFORE	1.0000
control II 3-h after - control I JUST AFTER	<0.001
control II 3-h after - suppl I JUST AFTER	<0.001
control II 3-h after - control II JUST AFTER	1.0000
control II 3-h after - suppl II JUST AFTER	1.0000
control II 3-h after - control III JUST AFTER	1.0000
control II 3-h after - suppl III JUST AFTER	1.0000
suppl II 3-h after - control III 3-h after	0.7228
suppl II 3-h after - suppl III 3-h after	1.0000
suppl II 3-h after - control I BEFORE	<0.001
suppl II 3-h after - suppl I BEFORE	<0.001
suppl II 3-h after - control II BEFORE	1.0000
suppl II 3-h after - suppl II BEFORE	<0.001
suppl II 3-h after - control III BEFORE	1.0000

suppl II 3-h after - suppl III BEFORE	1.0000
suppl II 3-h after - control I JUST AFTER	<0.001
suppl II 3-h after - suppl I JUST AFTER	<0.001
suppl II 3-h after - control II JUST AFTER	1.0000
suppl II 3-h after - suppl II JUST AFTER	1.0000
suppl II 3-h after - control III JUST AFTER	1.0000
suppl II 3-h after - suppl III JUST AFTER	1.0000
control III 3-h after - suppl III 3-h after	1.0000
control III 3-h after - control I BEFORE	<0.001
control III 3-h after - suppl I BEFORE	<0.001
control III 3-h after - control II BEFORE	0.0602
control III 3-h after - suppl II BEFORE	<0.001
control III 3-h after - control III BEFORE	0.0407
control III 3-h after - suppl III BEFORE	0.0155
control III 3-h after - control I JUST AFTER	<0.001
control III 3-h after - suppl I JUST AFTER	<0.001
control III 3-h after - control II JUST AFTER	1.0000
control III 3-h after - suppl II JUST AFTER	0.2316
control III 3-h after - control III JUST AFTER	1.0000
control III 3-h after - suppl III JUST AFTER	1.0000
suppl III 3-h after - control I BEFORE	<0.001
suppl III 3-h after - suppl I BEFORE	<0.001
suppl III 3-h after - control II BEFORE	1.0000
suppl III 3-h after - suppl II BEFORE	<0.001
suppl III 3-h after - control III BEFORE	1.0000
suppl III 3-h after - suppl III BEFORE	0.6140
suppl III 3-h after - control I JUST AFTER	<0.001
suppl III 3-h after - suppl I JUST AFTER	<0.001
suppl III 3-h after - control II JUST AFTER	1.0000
suppl III 3-h after - suppl II JUST AFTER	1.0000
suppl III 3-h after - control III JUST AFTER	1.0000
suppl III 3-h after - suppl III JUST AFTER	1.0000
control I BEFORE - suppl I BEFORE	1.0000
control I BEFORE - control II BEFORE	<0.001
control I BEFORE - suppl II BEFORE	0.9130
control I BEFORE - control III BEFORE	<0.001
control I BEFORE - suppl III BEFORE	<0.001
control I BEFORE - control I JUST AFTER	1.0000
control I BEFORE - suppl I JUST AFTER	1.0000
control I BEFORE - control II JUST AFTER	<0.001
control I BEFORE - suppl II JUST AFTER	<0.001
control I BEFORE - control III JUST AFTER	<0.001
control I BEFORE - suppl III JUST AFTER	<0.001
suppl I BEFORE - control II BEFORE	<0.001
suppl I BEFORE - suppl II BEFORE	1.0000
suppl I BEFORE - control III BEFORE	<0.001
suppl I BEFORE - suppl III BEFORE	<0.001
suppl I BEFORE - control I JUST AFTER	1.0000
suppl I BEFORE - suppl I JUST AFTER	1.0000
suppl I BEFORE - control II JUST AFTER	<0.001
suppl I BEFORE - suppl II JUST AFTER	<0.001
suppl I BEFORE - control III JUST AFTER	<0.001
suppl I BEFORE - suppl III JUST AFTER	<0.001
control II BEFORE - suppl II BEFORE	<0.001
control II BEFORE - control III BEFORE	1.0000
control II BEFORE - suppl III BEFORE	1.0000
control II BEFORE - control I JUST AFTER	<0.001
control II BEFORE - suppl I JUST AFTER	<0.001

control II BEFORE - control II JUST AFTER	1.0000
control II BEFORE - suppl II JUST AFTER	1.0000
control II BEFORE - control III JUST AFTER	1.0000
control II BEFORE - suppl III JUST AFTER	1.0000
suppl II BEFORE - control III BEFORE	<0.001
suppl II BEFORE - suppl III BEFORE	<0.001
suppl II BEFORE - control I JUST AFTER	1.0000
suppl II BEFORE - suppl I JUST AFTER	1.0000
suppl II BEFORE - control II JUST AFTER	<0.001
suppl II BEFORE - suppl II JUST AFTER	<0.001
suppl II BEFORE - control III JUST AFTER	<0.001
suppl II BEFORE - suppl III JUST AFTER	<0.001
control III BEFORE - suppl III BEFORE	1.0000
control III BEFORE - control I JUST AFTER	<0.001
control III BEFORE - suppl I JUST AFTER	<0.001
control III BEFORE - control II JUST AFTER	1.0000
control III BEFORE - suppl II JUST AFTER	1.0000
control III BEFORE - control III JUST AFTER	1.0000
control III BEFORE - suppl III JUST AFTER	1.0000
suppl III BEFORE - control I JUST AFTER	<0.001
suppl III BEFORE - suppl I JUST AFTER	<0.001
suppl III BEFORE - control II JUST AFTER	1.0000
suppl III BEFORE - suppl II JUST AFTER	1.0000
suppl III BEFORE - control III JUST AFTER	0.4355
suppl III BEFORE - suppl III JUST AFTER	0.5564
control I JUST AFTER - suppl I JUST AFTER	1.0000
control I JUST AFTER - control II JUST AFTER	<0.001
control I JUST AFTER - suppl II JUST AFTER	<0.001
control I JUST AFTER - control III JUST AFTER	<0.001
control I JUST AFTER - suppl III JUST AFTER	<0.001
suppl I JUST AFTER - control II JUST AFTER	<0.001
suppl I JUST AFTER - suppl II JUST AFTER	<0.001
suppl I JUST AFTER - control III JUST AFTER	<0.001
suppl I JUST AFTER - suppl III JUST AFTER	<0.001
control II JUST AFTER - suppl II JUST AFTER	1.0000
control II JUST AFTER - control III JUST AFTER	1.0000
control II JUST AFTER - suppl III JUST AFTER	1.0000
suppl II JUST AFTER - control III JUST AFTER	1.0000
suppl II JUST AFTER - suppl III JUST AFTER	1.0000
control III JUST AFTER - suppl III JUST AFTER	1.0000

Table S3. Post-hoc test results for CK MM.

Comparison	p-value
control I 3-h after - suppl I 3-h after	1
control I 3-h after - control II 3-h after	<0.0001
control I 3-h after - suppl II 3-h after	<0.0001
control I 3-h after - control III 3-h after	<0.0001
control I 3-h after - suppl III 3-h after	<0.0001
control I 3-h after - control I before	1
control I 3-h after - suppl I before	1
control I 3-h after - control II before	<0.0001
control I 3-h after - suppl II before	1
control I 3-h after - control III before	<0.0001
control I 3-h after - suppl III before	<0.0001
control I 3-h after - control I just after	1
control I 3-h after - suppl I just after	1
control I 3-h after - control II just after	<0.0001
control I 3-h after - suppl II just after	<0.0001
control I 3-h after - control III just after	<0.0001
control I 3-h after - suppl III just after	<0.0001
suppl I 3-h after - control II 3-h after	<0.0001
suppl I 3-h after - suppl II 3-h after	<0.0001
suppl I 3-h after - control III 3-h after	<0.0001
suppl I 3-h after - suppl III 3-h after	<0.0001
suppl I 3-h after - control I before	1
suppl I 3-h after - suppl I before	1
suppl I 3-h after - control II before	<0.0001
suppl I 3-h after - suppl II before	1
suppl I 3-h after - control III before	<0.0001
suppl I 3-h after - suppl III before	<0.0001
suppl I 3-h after - control I just after	1
suppl I 3-h after - suppl I just after	1
suppl I 3-h after - control II just after	<0.0001
suppl I 3-h after - suppl II just after	<0.0001
suppl I 3-h after - control III just after	<0.0001
suppl I 3-h after - suppl III just after	<0.0001
control II 3-h after - suppl II 3-h after	1
control II 3-h after - control III 3-h after	0.26
control II 3-h after - suppl III 3-h after	1
control II 3-h after - control I before	<0.0001
control II 3-h after - suppl I before	<0.0001
control II 3-h after - control II before	1
control II 3-h after - suppl II before	<0.0001
control II 3-h after - control III before	1
control II 3-h after - suppl III before	1
control II 3-h after - control I just after	<0.0001
control II 3-h after - suppl I just after	<0.0001
control II 3-h after - control II just after	1
control II 3-h after - suppl II just after	1
control II 3-h after - control III just after	1
control II 3-h after - suppl III just after	1
suppl II 3-h after - control III 3-h after	0.72
suppl II 3-h after - suppl III 3-h after	1
suppl II 3-h after - control I before	<0.0001
suppl II 3-h after - suppl I before	<0.0001
suppl II 3-h after - control II before	1
suppl II 3-h after - suppl II before	<0.0001
suppl II 3-h after - control III before	1
suppl II 3-h after - suppl III before	1

suppl II 3-h after - control I just after	<0.0001
suppl II 3-h after - suppl I just after	<0.0001
suppl II 3-h after - control II just after	1
suppl II 3-h after - suppl II just after	1
suppl II 3-h after - control III just after	1
suppl II 3-h after - suppl III just after	1
control III 3-h after - suppl III 3-h after	1
control III 3-h after - control I before	<0.0001
control III 3-h after - suppl I before	<0.0001
control III 3-h after - control II before	0.06020
control III 3-h after - suppl II before	<0.0001
control III 3-h after - control III before	0.04070
control III 3-h after - suppl III before	0.01550
control III 3-h after - control I just after	<0.0001
control III 3-h after - suppl I just after	<0.0001
control III 3-h after - control II just after	1
control III 3-h after - suppl II just after	0.23
control III 3-h after - control III just after	1
control III 3-h after - suppl III just after	1
suppl III 3-h after - control I before	<0.0001
suppl III 3-h after - suppl I before	<0.0001
suppl III 3-h after - control II before	1
suppl III 3-h after - suppl II before	<0.0001
suppl III 3-h after - control III before	1
suppl III 3-h after - suppl III before	0.61400
suppl III 3-h after - control I just after	<0.0001
suppl III 3-h after - suppl I just after	<0.0001
suppl III 3-h after - control II just after	1
suppl III 3-h after - suppl II just after	1
suppl III 3-h after - control III just after	1
suppl III 3-h after - suppl III just after	1
control I before - suppl I before	1
control I before - control II before	<0.0001
control I before - suppl II before	0.91300
control I before - control III before	<0.0001
control I before - suppl III before	<0.0001
control I before - control I just after	1
control I before - suppl I just after	1
control I before - control II just after	<0.0001
control I before - suppl II just after	<0.0001
control I before - control III just after	<0.0001
control I before - suppl III just after	<0.0001
suppl I before - control II before	<0.0001
suppl I before - suppl II before	1
suppl I before - control III before	<0.0001
suppl I before - suppl III before	<0.0001
suppl I before - control I just after	1
suppl I before - suppl I just after	1
suppl I before - control II just after	<0.0001
suppl I before - suppl II just after	<0.0001
suppl I before - control III just after	<0.0001
suppl I before - suppl III just after	<0.0001
control II before - suppl II before	0.00002
control II before - control III before	1
control II before - suppl III before	1
control II before - control I just after	<0.0001
control II before - suppl I just after	<0.0001
control II before - control II just after	1

control II before - suppl II just after	1
control II before - control III just after	1
control II before - suppl III just after	1
suppl II before - control III before	0.00003
suppl II before - suppl III before	<0.0001
suppl II before - control I just after	1
suppl II before - suppl I just after	1
suppl II before - control II just after	<0.0001
suppl II before - suppl II just after	<0.0001
suppl II before - control III just after	<0.0001
suppl II before - suppl III just after	<0.0001
control III before - suppl III before	1
control III before - control I just after	<0.0001
control III before - suppl I just after	<0.0001
control III before - control II just after	1
control III before - suppl II just after	1
control III before - control III just after	1
control III before - suppl III just after	1
suppl III before - control I just after	<0.0001
suppl III before - suppl I just after	<0.0001
suppl III before - control II just after	1
suppl III before - suppl II just after	1
suppl III before - control III just after	0.44
suppl III before - suppl III just after	0.56
control I just after - suppl I just after	1
control I just after - control II just after	<0.0001
control I just after - suppl II just after	<0.0001
control I just after - control III just after	<0.0001
control I just after - suppl III just after	<0.0001
suppl I just after - control II just after	<0.0001
suppl I just after - suppl II just after	<0.0001
suppl I just after - control III just after	<0.0001
suppl I just after - suppl III just after	<0.0001
control II just after - suppl II just after	1
control II just after - control III just after	1
control II just after - suppl III just after	1
suppl II just after - control III just after	1
suppl II just after - suppl III just after	1
control III just after - suppl III just after	1

Table S4 R2 for models

Effect	IL-10			IL-2/IL-10			CK MM		
	Rsq	upper CL	lower CL	Rsq	upper CL	lower CL	Rsq	upper CL	lower CL
Model	0.74	0.80	0.69	0.43	0.57	0.36	0.84	0.88	0.81
time point	0.71	0.77	0.64	0.15	0.27	0.06	0.82	0.86	0.78
effects of physical exercise testing:time point	0.14	0.27	0.07	0.31	0.43	0.21	0.12	0.24	0.05
effects of physical exercise testing	0.11	0.22	0.04	0.06	0.15	0.01	0.22	0.35	0.12
group	0.11	0.45	0.00	0.001	0.25	0.00	0.07	0.40	0.00
group:effects of physical exercise testing:time point	0.09	0.20	0.03	0.05	0.16	0.02	0.12	0.25	0.05
group:effects of physical exercise testing	0.01	0.08	0.00	0.004	0.06	0.00	0.04	0.13	0.01
group:time point	0.01	0.08	0.00	0.04	0.13	0.01	0.12	0.24	0.05
Model	0.74	0.80	0.69	0.43	0.57	0.36	0.84	0.88	0.81

Rsq-R squared, upper CL-upper confidence level, lower CL-lower confidence level.
